# Evidence on the economic value of end-of-life and palliative care interventions: a narrative review of reviews

**DOI:** 10.1186/s12904-021-00782-7

**Published:** 2021-06-23

**Authors:** Xhyljeta Luta, Baptiste Ottino, Peter Hall, Joanna Bowden, Bee Wee, Joanne Droney, Julia Riley, Joachim Marti

**Affiliations:** 1 Centre for Primary Care and Public Health (Unisanté), University of Lausanne, Route de la Corniche 10, CH-1010 Lausanne, Switzerland; 2 Institute of Global Health Innovation, Department of Surgery and Cancer, Imperial College London, London, UK; 3 Edinburgh Cancer Research Centre, University of Edinburgh, Edinburgh, UK; 4 NHS Fife, Scotland, UK; 5 University of St Andrews, Scotland, UK; 6 Harris Manchester College, University of Oxford, Oxford, UK; 7 The Royal Marsden NHS Foundation Trust, London, UK

**Keywords:** End-of-life care, Terminal care, Palliative care, Cost - effectiveness, Health care costs

## Abstract

**Background:**

As the demand for palliative care increases, more information is needed on how efficient different types of palliative care models are for providing care to dying patients and their caregivers. Evidence on the economic value of treatments and interventions is key to informing resource allocation and ultimately improving the quality and efficiency of healthcare delivery. We assessed the available evidence on the economic value of palliative and end-of-life care interventions across various settings.

**Methods:**

Reviews published between 2000 and 2019 were included. We included reviews that focused on cost-effectiveness, intervention costs and/or healthcare resource use. Two reviewers extracted data independently and in duplicate from the included studies. Data on the key characteristics of the studies were extracted, including the aim of the study, design, population, type of intervention and comparator, (cost-) effectiveness resource use, main findings and conclusions.

**Results:**

A total of 43 reviews were included in the analysis. Overall, most evidence on cost-effectiveness relates to home-based interventions and suggests that they offer substantial savings to the health system, including a decrease in total healthcare costs, resource use and improvement in patient and caregivers’ outcomes. The evidence of interventions delivered across other settings was generally inconsistent.

**Conclusions:**

Some palliative care models may contribute to dual improvement in quality of care via lower rates of aggressive medicalization in the last phase of life accompanied by a reduction in costs. Hospital-based palliative care interventions may improve patient outcomes, healthcare utilization and costs. There is a need for greater consistency in reporting outcome measures, the informal costs of caring, and costs associated with hospice.

## Background

In the context of increasing pressures on health budgets, evidence on the economic value of treatments and interventions is key to informing resource allocation and improving the quality and efficiency of healthcare delivery. As an important share of healthcare expenditures occurs in the last months of life [1–3], a good understanding of the costs and benefits of care delivered during this period is particularly important. Whether dying patients are receiving “appropriate” care has been the focus of recent debates [4, 5]. Notably, there are concerns that many patients are admitted to the hospital and undergo invasive procedures at the end-of-life without evidence of clinical benefits and improved quality of life (QoL); more than 80% of decedents are hospitalized at least once in the last 180 days of life in several countries [6]. In addition, research shows that such treatments are often counter to patient and/or caregiver preferences [7, 8]. Palliative and end-of-life care services and interventions have the potential to improve the quality, appropriateness and efficiency of care provided at the end-of-life from both the perspective of patients and their families and the health system as a whole [9]. Palliative care has been associated with improved patient outcomes, such as pain and symptom management [10], improved communication [11], higher satisfaction with care [12], improved QoL [13], reduced healthcare costs [14, 15] and an increased likelihood of dying in one’s preferred place [16]. Additionally, evidence suggests that such models of care could reduce healthcare resource use, including emergency hospital admissions, length of hospital stays (LOS) and ICU admissions [17–19].

As the demand for palliative care services increases, more information is needed on how effective and efficient different types of palliative care models are for providing care to patients and their caregivers. In particular, high-quality economic evidence regarding end-of-life and palliative care interventions is crucial to adequately support and develop new models of care that have the potential to improve the experience of patients and their relatives, avoid unnecessary treatments, and potentially reduce healthcare costs. Prior research has shown that the quality of economic evidence in the area is mixed [20]; it is often limited to assessments of reduced healthcare utilization, without proper measurement of intervention costs and/or valuation of benefits [21]. While several reviews on the topic of palliative care have been published in recent years, few have assessed the effectiveness and cost-effectiveness of palliative care interventions. Existing reviews have mainly focused on particular populations (e.g., cancer) [22], specific interventions (e.g., advance care planning, ACP) [23], or settings (e.g., home-setting) [24]. A recent review of international evidence provided important insights into a wide range of palliative care models. It found that irrespective of setting or patient characteristics, palliative care seem to be beneficial to the patients and may reduce total healthcare costs [25]. However, a more detailed assessment of effectiveness and cost-effectiveness has not been identified.

The aim of this systematic review is to provide a comprehensive overview of the available evidence published between 2000 and 2019 on the economic value of palliative and end-of-life care interventions across various settings (i.e., community, home, hospital, etc.). Due to growing evidence from multiple systematic reviews, a review of reviews is considered the most appropriate approach for bringing together the available evidence together on different interventions [26, 27].

We focused our search on published reviews that contained at least some economic evidence, including measures of cost-effectiveness, intervention costs and/or impact on healthcare use. Such knowledge might be valuable to support health policy makers in making resource allocation decisions and commissioning palliative care services.

## Methods

This systematic review was performed according to PRISMA guidelines (Preferred Reporting Items for Systematic Reviews and Meta-Analyses) [28]. This review is guided by Smith et al [26] methodology for conducting systematic reviews of systematic reviews. This review is registered in PROSPERO (registration number: CRD42018110910).

### Search strategies

We performed systematic searches using Ovid in the following databases: Medline, Embase, PubMed, Cinahl, Psychinfo, Scisearch and Cochrane. We searched reviews published between January 2000 and September 24, 2019, using both MESH and keywords in the fields of palliative and end-of-life care and health economics (Table 1). Reference lists of included reviews were screened for relevant studies. We also performed Google Scholar searches using key search terms (‘systematic review’, ‘palliative care’, ‘cost-effectiveness’).

**Table 1:** Search Strategy

Ovid MEDLINE(R) Epub Ahead of Print, In-Process & Other Non-Indexed Citations, Ovid MEDLINE(R) Daily and Ovid MEDLINE(R) < 1946 to Present>	
#	Search Strategy
1	exp Terminal Care/ or exp. Palliative Care/ or exp. Terminally Ill/ or ((End adj2 life adj2 care) or EoL care or (terminal* adj2 (care or caring or ill* or disease*)) or palliat* or dying or (Advanced adj3 (disease* or illness*)) or end stage*).ti,ab,kf.
2	(cost: or cost benefit analys: or health care costs).mp.
3	((exp Review Literature as Topic/ or exp. Review/ or (literature adj3 review*).ab,ti.) and ((medline or medlars or embase or pubmed or cinahl or amed or psychlit or psyclit or psychinfo or psycinfo or scisearch or cochrane).ab,ti. or Retracted Publication.pt.)) or Meta-Analysis as Topic/ or Meta-analysis.pt. or (systematic* adj2 (review* or overview)).ab,ti. or ((meta adj1 anal*) or metaanal* or metanal*).ab,ti.
4	1 and 2 and 3
5	limit 4 to (english language and yr = “2000 -Current”)

### Study selection

We included systematic reviews focusing on quantitative assessments of intervention effectiveness that contained economic evidence on cost-effectiveness, intervention costs and/or resource use. We included interventions related to palliative and end-of-life care without restriction of the setting or comparator definition. Following Brereton et al. (2017) [25], palliative and end-of-life care models/interventions were defined as “any structured care model involving multiple components, including who delivers (e.g., professionals, caregivers) the intervention (specialist or generalist palliative care), where (e.g., hospital), to whom (care recipients), when (i.e., timing and duration), how (e.g., face to face) and for what purpose (i.e., expected outcomes).” Details on inclusion and exclusion criteria are reported in Table 2.

**Table 2:** Inclusion and exclusion criteria

Criteria	Inclusion	Exclusion
Type of study	Review-level (review of reviews) evidence focusing on quantitative assessment effectiveness in palliative care interventions. Reviews of all types of original studies; reviews of reviews.	Not review-level evidence (primary studies). Opinion papers, editorials, conference abstracts. Reviews of qualitative studies.
Dates	2000–2019	Outside the date range 2000–2018
Population	Reviews considering terminally ill adults (18 years old and over) and considering patients with varying illnesses.	Reviews focusing solely on children and adolescents (under 18 years of age) Reviews focusing on specific populations or ethnicities.
Intervention	Reviews considering interventions in palliative care for any palliative care group.	Reviews not focusing on palliative care. Reviews focusing on a single procedure (i.e., a focus on treatment rather than palliative care) such as chemotherapy, radiotherapy, surgery and other curative strategies. Reviews focusing on palliative care, but not on interventions. Reviews focusing on treatment rather than palliative care.
Economic outcomes: Information on cost-effectiveness and costs	Reviews including any information regarding costs, whether cost, cost for caregivers cost of intervention, hospitalization, or general costs at end-of-life.	Reviews not including quantitative cost elements.
Other outcomes	Resource use	–
Language	Written in English.	Not written in English.

### Study selection

Two reviewers (BO, XL) screened independently and in duplicate all titles and abstracts using the inclusion and exclusion criteria (Table 1). A third reviewer (JM) provided arbitration in the event of disagreement. The same reviewers assessed the full texts of potentially relevant for eligibility. Any disagreement was resolved by discussion and by consulting the third reviewer.

### Assessment of quality of included studies

We assessed the methodological quality of included studies using the AMSTAR. We assessed the methodological quality of included studies using the AMSTAR 2 (A Measurement Tool to Assess Reviews) which is a critical appraisal tool for systematic reviews that include randomized or non-randomised studies of healthcare interventions, or both [29]. The reviews were rated as low, medium quality or high quality.

### Data extraction

Two authors (XL, BO) extracted data using a predefined extraction form, and any discrepancies were resolved by a third reviewer. Data on the key characteristics of the studies were extracted, including information about the aim of the review, design, population, type of intervention and comparator, information on (cost) effectiveness and resource use, and main findings and conclusions.

### Data synthesis and analysis

The reviews were too heterogeneous in terms of disease, setting and outcomes measured to perform a meta-analysis; a narrative synthesis was considered more appropriate. We organized the evidence according to the main outcomes reported, i.e.: (1) cost-effectiveness, (2) costs, and (3) resource use. In each section, we discuss evidence by intervention setting and/or type.

## Results

**Fig. 1:**
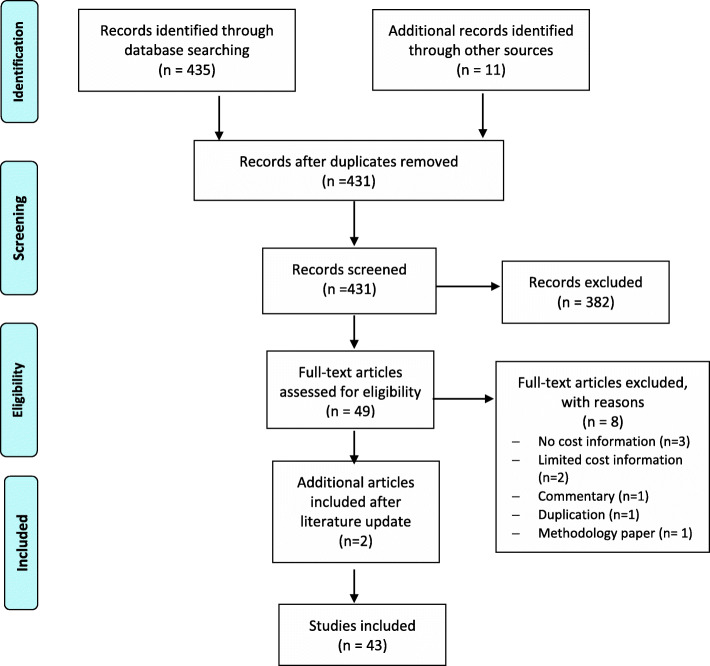
PRISMA flowchart

The initial searches identified 435reviews. Finally, 43 review articles were included in the analysis. Figure 1 provides details on searches and study selection. Over half of the reviews (Table 3) were published between 2011 and 2018 and included predominantly cancer patients. A wide range of palliative and end-of-life care models were described and comparator models were often described as usual care, which varied across studies, or was not specified. Most of the reviews (*n* = 21) involved interventions delivered in multiple settings [20–23, 25, 30–50]. Nine reviews examined interventions in the hospital setting [20, 50–58]. Of these, 3 focused on ICU [51–53], and 1 on surgical patients [57]. Three reviews involved home-based interventions [24, 57, 59, 60], and 3 reviews focused on community-based interventions [61–63]. Most of the included reviews were rated as having a moderate quality (Table 3).

**Table 3:** Characteristics of the included studies

Author, type of review	Aim	Type and number of studies	Population	Intervention	Comparator model	Outcomes	Key conclusions	Quality assessment (AMSTAR score)
Ankuda, and Meier., 2018 Review	To examine interventions and policies associated with high-value end of-life care	13 cohort studies, 1 meta-analysis,2 RCTs	Seriously ill population who have a high risk of dying but are not necessarily near the end of life	Community-based, ACP, home care, hospice care, home-based, public policy to create a new hospice benefit, nursing home–hospice collaboration, public financing of medical and long-term care, inpatient palliative care	Usual care	Costs, place of death, hospital stays, ACP, hospice, ICU/Invasive therapies	The efficacy of a range of approaches to improve value of care at the end of life, both within the health system and across public policy sectors	Moderate
Bainbridge and Sussman., 2016 Systematic review	To determine which components of in-home EoL programs are most commonly associated with better outcomes than usual care	17 systematic reviews, 2 narrative reviews	Individuals in an advanced, palliative, or EoL stage of illness	In-home palliative care multicomponent interventions linkage with hospital, around-the-clock home visits, physician home visits, team contact on-call, etc.	Convential care	QoL (patient +caregiver), satisfaction with care (patient +caregiver), performance status, pain and other symptom management, place of death, reduction in healthcare use or costs	Moderate evidence for improved outcomes: individual and caregiver satisfaction, pain and nonpain symptom management, supporting home deaths and reduction in healthcare use and costs	Moderate
Bibas, L., et al.,2019 Systematic review	To determine the association of such interventions with patient- and family-centered outcomes and resource use	13 RCTs performed in ICU	Surrogate decision-makers or family members	Surrogate decision-making Interventions performed in ICU targeting	Usual care	Patient-related clinical outcomes, SDM and family-related outcomes (and use of resources (cost of care and health care resource use)	Surrogate decision-making for critically ill adults may reduce ICU LOS among patients who die in the ICU, without influencing overall mortality	High
Bradley et al., 2018 Systematic review	To summarize available evidence on the effectiveness and cost-effectiveness of palliative care interventions that facilitate social support	16 studies, including 3 using mixed methods 3 RCTs, 7 randomized prospective studies, 1 non-randomized prospective comparative study, 1 pilot study, 1 prospective study with matched comparison group, 1 quasi-experimental prospective comparative study, 1 prospective comparative study inpatient unit), 1 report on implementation of PROMS (no control)	Adults outpatients with a diagnosis of life-limiting (incurable) illness, including but not limited to cancer. Other diagnosis including lung disease, progressive neurological disease and kidney disease	Palliative care interventions offering opportunities for social support, facilitating face-to-face interactions with other people outside of the individual’s home	None/any	Perceived social support, QoL, psychological distress and symptoms. Emphasis on QoL outcomes	Weak evidence for improved QoL outcomes The majority of papers reported that the intervention improved psychological well-being (7 significant).	Moderate
Brereton et al., 2017 Systematic review and narrative review	To identify the existing range of models of palliative care that have been evaluated, and what further research is necessary to identify the most effective and cost-effective palliative care models	18 reviews: meta-analyses, narrative reviews, and one meta-synthesis	Adults (over 18) with life-limiting illnesses. Mixed diagnosis (cancer, advanced cancer COPD, CHF, motor neurone disease, dementia, HIV/AIDS)	Models of palliative care for any palliative patient group	Usual care (e.g., standard, hospital, hospice care, primary care, usual oncology care), any alternative care, traditional long-term care etc	All outcomes reported in the original studies	Moderate evidence suggests that models of palliative care appear to show benefits and some models of palliative care may reduce total healthcare costs	Moderate
Brighton, L.J., et al.,2019 Systematic review and meta-analysis	To examine the outcomes, experiences and therapeutic components of Integrated palliative care these services	37 articles including randomised controlled trials (RCTs) and non-RCTs, observational studies and qualitative studies	People with advanced disease and chronic breathlessness	Information and education, psychosocial support, self-management strategies, other interventions	Usual care	Health outcomes, costs and utilization, quality-adjusted life-years (QALYs), patient and carer perspectives	Holistic services for chronic breathlessness can reduce distress in patients with advanced disease and may improve psychological outcomes of anxiety and depression	High
Candy et al., 2011 Systematic review	To identify the evidence on the effectiveness (including cost-effectiveness) of hospices and hospice care in a patient’s home and in nursing homes, and the experiences of the user/provider of these services	18 quantitative studies (including 2 RCTs and 6 cost evaluations), 4 qualitative studies	Terminally ill adults. Mostly cancer patients	EoL care service provided either at a dedicated hospice facility or at home, in a nursing home or other care facility in the community	Usual generalist healthcare	Symptom management, pain assessment, satisfaction with services, family carer well-being, health service use, costs and place of death. Patients’ emotional well-being (QoL, etc)	evidence suggest that hospice care may improve pain management and decrease hospital death. Many evaluations (particularly costs) are methodologically limited	Moderate
Datla, S., et al.,2019 A systematic review and narrative synthesis.	To identify the evidence in relation to palliative care for people with symptomatic heart failure	13 interventional and 10 observational studies	People with symptomatic heart failure	Multi disciplinary palliative care	Usual care	Patient-reported outcomes (symptom burden, depression, functional status, quality of life), resource use and costs of care	Multi-disciplinary palliative care in people with advanced heart failure but trials do not identify who would benefit most from specialist palliative referral	Moderate
Davis et al., 2015 Systematic review	To review and discuss RCTs examining the integration of palliative care earlier in the course of the disease trajectory for patients with serious illnesses as an outpatient and at home	15 RCTs of outpatient palliative care, 13 RCTs of home palliative care, 7 systematic reviews	Mostly advanced cancer patients. Other diagnosis: advanced COPD and heart failure, motor neuron disease, cancer, AIDS	Early integration of outpatient and home palliative care	Usual or conventional care	Wide range of outcomes reported. Main focus on costs, QoL, resource utilization, aggressiveness of care	Moderate evidence showing several benefits to early outpatient palliative care for patients with newly diagnosed metastatic cancer. Methodological issues account for differences in results	Moderate
Dixon et al., 2015 Systematic review	To review and summarize economic evidence on ACP	18 studies. 4 RCTs, 1 cluster-RCT, 13 observational studies	Patients aged over 18	ACP, defined as including advance directives/decisions, advance care statements or written plans, and/or ACP discussions	Comparison between people engaging and not engaging in ACP	Economic outcomes. 10 studies include primarily hospital costs. Non- healthcare costs omitted from analyses. Intervention costs not always reported. No health-related QoL measures	Moderate evidence that ACP is associated with healthcare savings (statistically significant in half of the studies). There is a need to consider wider costs including cost of intervention and the costs of substitute health, social and informal care	Moderate
Dixon et al., 2018 Systematic review	To review the evidence concerning the effectiveness of ACP in improving EoL outcomes for people with dementia and their carers	13 studies: 3 RCTs	People with dementia and their carers	ACP	Unclear	Health utilization (stay, and hospital cost and patient and carer outcomes	Low evidence regarding ACP.ACP is likely to be relevant to dementia patients and in certain circumstances is associated with positive EoL outcomes	Moderate
Douglas et al., 2003 Systematic review	To assess how the measurement of economic outcomes has been tackled in the literature	17 studies were included: 6 RCTs, 4 before-and-after studies, 3 quasi-experimental studies, 1 randomized crossover trial, 2 retrospective studies, 1 case study	Cancer, non-cancer, paediatrics, obstetrics	Clinical nurse specialist cancer (CNSs) and palliative nursing	When reported, standard care, hospital care, consultant, usual care	Costs, resource use, patient complications, QoL, outcomes related to specific interest to nurses	CNSs were reported to be less costly and more effective than alternative care. Overall, the evidence was low. Higher-quality economic evaluations are needed	Moderate
El-Jawahri et al., 2011 Systematic review	To review the efficacy of palliative care interventions patients with incurable disease	5 RCTs were included	Patients with incurable disease	Various palliative care inteventions with palliative care focus	Standard care, usual care, standard follow-up. Not receiving intervention, managed care	QOL, physical and psychological symptoms, family caregiver outcomes, satisfaction with care, health-services utilization, and end-of-life outcomes	Low evidence that palliative care interventions do improve patients’ quality of life, satisfaction with care, and end-of-life outcomes.	Moderate
Francke and Anneke, 2000 Systematic review	To review evidence on the effectiveness of palliative support teams	16 evaluative studies were included	Patients receiving palliative care	Palliative care support teams	Unclear	Consumption, and costs of healthcare, outcomes regarding physical, psychosocial or spiritual problems	Low evidence that palliative support teams reduce or increase care consumption and costs. Higher-quality research is needed	Moderate
Garcia-Perez et al., 2009 Narrative review	To report evidence on the effectiveness and cost-effectiveness of specialized palliative care programs for terminally ill patients	6 reviews, 3 studies on effectiveness and 1 cost study	Adults (18 years and older) with terminal illness (cancer and other diseases) included in a palliative care programme	Specialized palliative care programs. Full-PCT, telephone-PCT, home−/hospital-based hospices, small specialist palliative care unit, telehospice	Comparing palliative care programs in adults against one another	Control of symptoms at one week, satisfaction of patients and carers, QoL (EORTC QLQ-C30), pain control, number of inpatient days, number of home visits, place of death, total cost per patient	Low evidence I on the effectiveness and cost-effectiveness of different models, further research of high quality is needed	Moderate
Gleeson, et al., 2019 Systematicreview	To identify the most effective ACP interventions to train/educate all levels of healthcare professionals working in care homes	Three before and after studies, 1 cluster randomised controlled trial (RCT), 1non-blinded RCT and one qualitative study	Healthcare professionals working in care home setting	Advance care planning for home health staff	No intervention, usual care, and comparison within groups	Health-related outcomes, hospitalisation rate, days and healthcare costs; hospital deaths	There is limited evidence for the effectiveness of ACP training for care home workers.	Moderate
Gomes et al., 2013 Systematic review, meta-analysis and narrative synthesis	To quantify the effect of home palliative care services for adult patients with advanced illness and their family caregivers	23 studies were included: 16 RCTs, 4 CCTs (including 2 cluster CCTs), 2 CBAs, 1 ITS	Participants aged 18 years or older in receipt of a home palliativecare service, their family caregivers, or both.symptomatic, or both	A team delivering home palliative care 1) for patients with a severe or advanced disease or their caregivers 2) aiming to support patients and family caregivers, and enable them to stay at home 3) provided specialist or intermediate palliative/hospice care and 4) providing comprehensive care	Home palliative care vs usual care, home vs hospital palliative care	Primary: death at home, Secondary: time spent at home, satisfaction with care, pain and other symptoms, physical function, QoL, caregiver outcomes and costs	Meta-analysis showed increased odds of dying at home, narrative synthesis showed evidence of statistically significant beneficial effects of home palliative care vs usual in reducing symptom burden. Evidence on cost-effectiveness is moderate	High
Harris and Murray, 2013 Systematic review	To review the evidence for palliative interventions reducing health service costs without impacting on quality of care	12 studies were included: 6 RCTs, 2 prospective cohort studies, and 4 retrospective case-controlled studies	Patients thought to be in their final year, months or weeks of life. Mixed diagnosis. When reported, cancer, COPD, heart failure, AIDS	A consciously palliative approach to terminal care	Palliative care intervention vs the routine package of care prevailing in the health service under scrutiny	Financial cost, and patient QoL or satisfaction with care	Moderate evidence that palliative care interventions generally reduce health service costs. Evidence of concurrent improvement in QoL outcomes waslow. Small sample sizes and disparate outcome measures hamper statistical assessments	Moderate
Higginson and Evans, 2010 Systematic review (meta-synthesis)	To determine whether specialist palliative care teams improve outcomes for patients with advanced cancer and their caregivers	40 were included: 8 RCTS, 32 observational studies (usually with a control group)	Cancer patients	Palliative care teams at home, hospital or designated inpatient settings	Usual care (present or historical). Conventional community and general hospital/oncology services	Pain and symptom management, QoL and death, and patient and carer satisfaction/morbidity before and after bereavement	High evidence that home, hospital, and inpatient specialist palliative care significantly improved patient outcomes in the domains of pain and symptom control, anxiety, and reduced hospital admissions	High
Higginson et al., 2003 Systematic review (meta-regression, meta-synthesis	To determine the effectiveness of palliative and hospice care teams	44 studies were included	Patients with a progressive life-threatening illness and their caregivers (defined as family, friends, or significant others)	Palliative and hospice care teams	Usual care (routine community and general hospital/oncology services)	Pain and symptom control, quality of life and death; patient and family satisfaction/morbidity pre- and post-bereavement	This review shows a quantitative benefit to patients from the intervention of palliative care teams.	High
Higginson et al., 2002 Systematic review (qualitative meta-synthesis and quantitative meta-analysis	To determine whether hospital-based palliative care teams improve the process or outcomes of care for patients and families at the EoL	44 studies were included (1 RCT)	Patients with a progressive life-threatening illness, and their family, carers, or close friends	Hospital-based palliative care teams	Usual care (routine community and general hospital/oncology services, and isolated professionals who have undertaken limited training in palliative care)	Pain, control of other specific symptoms such as nausea, anorexia, tiredness, improved quality of life and quality of death, patient satisfaction and carer satisfaction pre-bereavement, carer morbidity pre- and post-bereavement	Low evidence that hospital-based palliative care teams offer some benefits. There is a need for higher-quality research	High
Khandelwal et al., 2005 Systematic review	To assess the effects of ACP and palliative care interventions on ICU admissions and length of stay	22 studies were included: 9 RCTs and 13 non-randomized controlled trials	Critically ill adult patients (over 18)	Interventions inclusive of ACP, primary and specialty palliative care, and ethics consultation that include a focus on the goals of care. Outpatient, acute care and ICU settings	Palliative care vs usual care	ICU length of stay and ICU admission	High evidence ACP or palliative care interventions consistently showed a pattern toward decreased ICU admissions and reduced ICU LOS. Provides a basis for modeling impact on healthcare costs	Modearte
Klingler et al., 2016 Systematic review	To describe the cost implications of Advance Care Planning programs and discuss ethical conflicts arising in this context	7 studies were included: 4 RCTs, 1 CBA, 2 observational studies	All patient groups	Any intervention involving a communication process facilitated by a professional caregiver involving the patient and/or legal proxy about the patient’s preferences for future medical care	Any intervention as comparator	Healthcare costs and cost-effectiveness. Excluded studies with indicators like ICU length of stay because they do not provide an account of the net resource use resulting from ACP interventions	Low evidence indicate net costs savings may be realized with advance care planning.	Moderate
Kyeremateng et al., 2018 Narrative review	To evaluate the effect of palliative care consultations in the ICU on length of stay (LOS) and costs	8 studies were included: 1 RCT, 4 retrospective cohort studies, 2 comparative studies of retrospective and prospective cohorts, and 1 prospective pre/post control group trial.	Adult medical patients (various diseases) receiving palliative care within the ICU	Palliative care services within the intensive care unit	Usual care	Primary: ICU LOS. Secondary: mortality, hospital LOS, and costs	Low evidence shows trend that PC consultations reduce LOS and costs without impacting mortality	Moderate
Lilley et al., 2016 Systematic review	To characterize the content, design, and results of interventions to improve access to palliative care or the quality of palliative care for surgical patients	25 studies were included: 9 single-institution retrospective cohort studies, 7 single-institution prospective cohort studies, 7 single-institution RCTs, and 2 multi-center RCTs	Adult patients, more than 20% surgical patients	Palliative care interventions focusing on surgical patients	Usual care	Palliative care consultation, ACP discussions, symptom burden, QoL, patient or caregiver satisfaction, quality communication, use and cost of healthcare services, and mortality	Although most of the studies reported positive findings, the evidence regarding usefulness of palliative care interventions in surgical patients is moderate Higher-quality research is needed	Moderate
Luckett et al., 2013 Systematic review and meta-analysis	To examine whether community specialist palliative care services (SPCSs) offering home nursing increase rates of home death compared with other models	10 studies (RCTs and non-RCTs)	Adults with life-limiting illnesses	Community-based SPCS providing home nursing	Usual care (not receiving the intervention) when specified	Primary: place of death. Secondary: symptom control, QoL and costs	Moderate evidence that SPCSs offering home nursing increase home deaths without compromising symptoms or increasing costs but there is a compelling trend	High
Martin et al., 2016 Systematic review	To identify the effects of ACP interventions on nursing home residents	13 studies were included: 1 RCT, 5 controlled trials, 5 prospective cohorts, 2 pre-post interventions	Nursing home residents	ACP	Not clearly described. Where stated, usual care, not receiving the intervention.	Hospitalization and costs, place of death, resident’s wishes, use of life-sustaining treatments, QoL and satisfaction, mortality, in-patient hospice and community palliative care, DNR orders and interventions	Moderate evidence suggests that ACP has beneficial effects in nursing home residents. Type of interventions and outcomes vary between studies which makes it difficult to identify the effectiveness of one intervention over another	Moderate
May et al., 2018 Meta-review	To review the economic evidence on specialist palliative care consultation teams in the hospital setting	10 studies were included: 9 cohort studies and 1 RCT	Adults patients in hospital setting	Specialist-led multidisciplinary palliative care consultation	Usual care	Overall costs, ancillary costs, ICU costs	Moderate evidence suggests that specialist palliative care teams less costly and improve care of patients with serious illness compared to usual care	Modearte
May et al., 2014 Meta-analysis	To estimate the association of palliative care consultation with direct hospital costs for adults with serious illness	6 cohort studies	Adults (cancer, heart, liver, or kidney failure; COPD; AIDS/HIV; or selected neurodegenerative conditions) in the hospital setting	Palliative care consultation	Usual care	Total direct hospital costs	Modearte evidence that palliative care consultation of hospitalization may reduce cost of care for hospitalized adults with life-limiting illness. Estimates may be larger for cancer and more morbidities compared to non-cancer patients and those with fewer morbidities	Moderate
Meads, D.M., et al.2019 Meta-analysis	To evaluate effectiveness of pain management interventions	RCTs	Patients with advanced cancer	Pain self-management interventions	Usual care	Cost-effectiveness	Educational and monitoring/feedback interventions have the potential to be cost-effective.	High
Oczkowski et al., 2016 Systematic review and meta-analysis	To determine the impact of communication tools for EoL decision-making in the ICU	19 studies were included: 4 RCTs, 1 cluster RCT, 14 cohort studies	Patients 18 years and over considered likely to die	Communication tools for EoL decision-making in the ICU	Usual care	Goals of care, code status decisions to withdraw or withhold life-sustaining treatments, patient or family satisfaction with EoL care, patient or family, knowledge about EoL care quality communication between the patient/ substitute decision-makers and healthcare providers, resource use, acceptability of the intervention	Communication tools may help improve documentation in EoL decision-making and may result in lower resource use. However, the evidence is low to very low quality	High
Rabow et al., 2013 Narrative review	To review and assess the evidence of the impact of outpatient palliative care	14 studies: 10 prospective RCTs, 4 prospective cluster RCTs	Patients receiving outpatient palliative care, their family caregivers, and their clinicians	Outpatient palliative care	Usual care	Patient, family and clinician satisfaction, symptom management, QoL and mortality, readmission rates, hospice use and costs	Moderate evidence supports the ongoing expansion of innovative outpatient palliative care service models throughout the care continuum to all patients with serious illness	Moderate
Salamanca-Balen et al., 2018 Systematic review	To review the evidence on costs, resource use and cost- effectiveness of Clinical Nurse Specialist–led interventions for patients with palliative care needs	79 studies were included: 37 RCTs, 22 quasi-experimental studies, 7 service evaluations and other studies, and 13 economic analyses	Adults aged 18 years and over with a clinical diagnosis of a life-limiting or life-threatening illness, who were unlikely to be cured, recover or stabilize	Clinical Nurse Specialist–led interventions	Not clearly described. Where stated, usual care	Costs, health system utilization such as length of stay (LOS), hospitalizations/ readmissions or health resource use (e.g. medications) and cost-effectiveness measures (e.g. incremental cost/ effectiveness ratios)	Moderate evidence suggest that clinical Nurse Specialist-led interventions for patients with palliative care needs may be effective in reducing resource use (hospitalizations/re-hospitalizations/admissions, length of stay) and healthcare costs	Moderate
Scheunemann et al., 2011 Systematic review	To investigate the effectiveness of communication interventions with regard to improving patient-or family-centered outcomes and reducing costs or resource use	21 articles of 16 distinct interventions were included: 5 RCTs, 3 non-RCTs, 9 prepost, 2 historical controls, 1 time-interrupted (A-B-A)	Patients in ICU aged 18 or older	Intervention to improve communication in intensive care	Usual care	Patient-and family-centered outcomes, costs and resource use	Printed information and structured communication can improve family comprehension and reduce ICU length of stay and treatment intensity. Low evidence on the impact of communication interventions on costs	Modearte
Shepperd et al., 2016 Systematic review	To determine if providing home-based EoL care reduces the likelihood of dying in hospital and what effect this has on patients’ symptoms, QoL, health service costs, and caregivers, compared with inpatient hospital or hospice care	4 RCTs were included	Adults aged 18 years and over, who are at the EoL and require terminal care	Home-based EoL providing active treatment for continuous periods of time by healthcare professionals to patients who otherwise require hospital or hospice inpatient EoL-care	Inpatient hospital or hospice care	Place of death and unplanned/precipitous admission to or discharge from hospital, control of symptoms, delay in care, participant health outcomes and patient satisfaction, family - or caregiver-reported symptoms, and health services cost and use	High evidence shows that people who receive EoL care at home are more likely to die at home. Future research should focus on the impact of home-based interventions on family members and lay caregivers	Moderate
Singer et al., 2016 Systematic review	To identify published evidence to inform how payers and providers should identify patients with advanced illnesses and specific interventions they should implement	124 RCTs were included	Adults 18 years or older with advanced illness, and/or their caregivers	Health service interventions addressing patient and/or caregiver quality-of-life-related elements in intervention design and/or as outcomes	Not clearly described. Where stated, usual care, did not receive care from palliative care team	Patient and caregiver quality-of-life-relevant outcomes. Economic outcomes: healthcare use and costs	Moderate evidence on costs and cost outcomes in palliative care. Palliative care interventions can improve the outcomes with the strongest evidence in cancer, CHF and COPD patients. Such models include nurse, social workers, and home-based components focusing on communication, psychosocial support as well as patient or caregiver experience	Moderate
Smith et al., 2014 Narrative review	To assess the available evidence on the costs and cost-effectiveness of palliative care interventions in any setting (e.g. hospital-based, home-based and hospice care)	46 studies were included: 5 RCTs, 2 non-RCTs, 34 cohort studies, 2 case studies. 2 before-and-after studies and 1 other study	Not clearly described. Cancer and non-cancer patients with advanced disease	Palliative care interventions across different settings (hospital-based, home-based, hospice care)	Usual care	Costs or resource use implications and cost-effectiveness	Moderate evidence suggests that palliative care is less expensive relative to comparator groups. In most cases the difference in cost was statistically significant	Moderate
Smith and Cassel, 2009 Brief review	To describe what is known about potential cost and other non-clinical outcomes (LOS) of palliative care compared with usual care	Number of included studies is unclear. RCT-s, non-RCTs	Patients receiving palliative care	Palliative care interventions	Usual care	Cost and non-clinical outcomes, length of stay, ICU	Moderate evidence on the impact of palliative care consultations, inpatient length of stay are related to local patterns of care	Moderate
Thomas et al., 2014 Systematic review	To assess the evidence on EOL case management	17 studies were included: 5 RCTs, 6 cohort studies, 4 non-randomized designs, 1 case study. Last one not mentioned	Palliative care patients including frail seniors chronically ill, cancer, advanced heart, lung, liver, or neurological disease, HIV, renal failure, advanced cancer	EOL case management	Not clearly described. Where stated, usual care, did not receive case management	Seeking to determine or establish the value of EOL case management (hospital utilization and additional value considerations) and identifying ways of improving EOL case management	t Moderate evidence suggests that case management may help reduce the need for hospital-based care and thus reduce healthcare costs	Moderate
Thomas and Sheps, 2006 Systematic review	To identify and analyze all published RCTs that focus on the organization of EoL care	23 RCTs were included	Terminally ill people near death, or dying, patients and family members	Community teams, specific palliative care interventions: advanced planning, patient-held records, providing QoL data, grief education, palliative-care education for nurses, care for dementia	Routine care, standard care, usual care, customary Veterans Affairs post-discharge care, conventional care, conventional hospital-based care, standard home care or office care, usual post-surgical care	QoL, symptom management, satisfaction with care, duration of palliative period, place of death, costs of palliative care compared to conventional care	Moderate evidence suggests that community or home-based EoL care can improve QoL and symptom management as well as opinions of patients and caregivers. Unclear whether community or home-based care is more cost-effective	Moderate
Totten et al., 2016 Systematic review	To assess the evidence about home-based primary care (HBPC) interventions for adults with serious or disabling chronic conditions	19 studies were included: 2 RCTs, 5 retrospective cohort, 5 prospective pre/post, 7 retrospective pre/post	Adults with chronic or disabilities	Home-based primary care	Any other model of primary care	Healthcare outcomes including mortality, morbidity and function. Patient and caregiver experience. Use of healthcare and costs	Moderate -evidence that HBPC reduces use of inpatient service, and low-strength evidence on the use of other healthcare services, cost and patient and caregiver experience	Moderate
Walczak et al., 2016 Systematic review	To identify and synthesize evidence for interventions targeting EoL communication and any or all stakeholders involved in communication	45 studies were included: 18 RCTs, 5 non-RCTs, 19 pre-post study, 3 post-only with control group or retrospective baseline data	Patients receiving EoL care, caregivers, health professionals across different clinical settings	Interventions targeting EoL communication	Usual care	Patient communication, healthcare professional communication, multifocal communication	The interventions have mainly targeted healthcare professionals in cancer patients. More research is needed targeting patients and caregivers. Moderate evidence that interventions targeting multiple stakeholders may be more effective in removing barriers to EoL communication.	Moderate
Waller et al., 2017 Systematic review	Examine the quantity and quality of data-based research aimed at improving: a) processes and (b) outcomes associated with delivering EoL care in hospital setting	18 intervention studies: 1 cluster randomized controlled trial, 1 stepped wedge trial, 14 RCTs, 2 (1 listed twice as RCT and controlled trial) controlled clinical trials and 1 interrupted time-series trial	Adults (18 years or over) admitted to hospitals (excluding intensive care units) or their families	EoL/goals of care discussions; EoL documentation (e.g. ACDs, DNR orders); appointment of substitute decision makers; medication orders; or referrals to hospice/palliative care	Compared different interventions with each other	Health status, satisfaction and quality of life, perceived quality of care, concordance of preferred and actual care, survival or healthcare costs or utilization	Moderate evidence reported benefits for end-of-life processes More methodologically robust studies are needed to evaluate the impact of interventions on EoL care outcomes.	Moderate
Zimmermann et al., 2008 Systematic review	Review the evidence on effectiveness of specialized palliative care in improving quality of life, satisfaction with care, and economic cost	22 RCTs: 4 used cluster randomization	Palliative care patients (cancer, congestive heart failure (CHF), COPD, motor neuron disease, and AIDS)	Specialized palliative care service that provides or coordinates comprehensive care to terminally ill patients	Usual care	Quality of life, satisfaction with care, economic cost and resource use	Moderate evidence to support the benefits of specialized palliative care interventions. Of three outcomes, there was consistent evidence only for caregiver satisfaction.	Moderate

### Evidence synthesis

#### Cost-effectiveness

In this section, we focus on studies that measured both the costs and intervention outcomes. Ideally, these components would be combined in an incremental cost-effectiveness framework, following best-practice economic evaluation guidelines [64]. Such evidence is rare [20, 21]; therefore, the evidence shown below (Table 4) also includes studies that measured both components independently. Overall, only 10 reviews included evidence on cost-effectiveness of the interventions, while the remaining 33 reviews included details on intervention costs and/or resource use.

**Table 4:** Studies reporting on cost-effectiveness of palliative care interventions

Author	Cost-effectiveness	Type of intervention
Ankuda, and Meier., 2018	Reported benefits of palliative care consultations on patient outcomes, use of healthcare and costs.	Hospital-based palliative care consultations
Bradley et al.,2018	Reported some evidence of cost-effectiveness of psychosocial support on survival in women with breast cancer. No significant difference in healthcare resource use between the intervention and control groups was found.	Clinical nurse specialists
Brereton et al., 2017	Various home-based interventions showed benefits for patients and caregivers and reduced healthcare costs.	Home-based
Candy et al., 2011	Hospice care interventions reduce resource use and costs, improve pain management, and increase death outside the hospital.	Hospice care at home, nursing homes and hospice facilities
Douglas et al., 2003	Reported lower costs and greater benefits of CNSs compared to usual care. Evidence of cost-effectiveness of pain management strategies in advanced cancer patients.	Clinical nurse specialists
Gomes et al., 2013	Reported clear cost-effectiveness of home-based interventions compared to usual care in 2 studies.	Home-based
Higginson et al., 2003	Evidence of the cost-effectiveness of home-based teams for specific patient groups including cancer and AIDS.	Home-based
Meads, D.M., et al.2019	Evidence from RCTs Educational and monitoring/feedback interventions have the potential to be cost-effective in patients with advanced cancer.	Hospital-based
Salamanca-Balen et al., 2018	Inconclusive evidence suggesting cost-effectiveness of CNS interventions including improvement in health economic outcomes, mainly in cardiac patients (heart failure) in Austria, UK and Netherlands.	Clinical nurse specialists
Smith et al., 2014	Reported one cost-effectiveness that showed short-term palliative care in multiple sclerosis patients showed d potential cost-effectiveness when caregiver burden was used as the main outcome measure.	Short-term palliative care

### Home-based interventions

Overall, most cost-effectiveness evidence relates to home-based interventions. One review [32] showed that home-based services may reduce resource use and costs and improve pain management and increase death outside the hospital.

Furthermore, home-based teams may generate substantial savings for the health system and improve patient outcomes. A review [47] identified 2 studies [65, 66] providing evidence of the cost-effectiveness of home-based teams for specific patient groups (i.e., cancer AIDS). A review [59] identified 6 studies of home-based palliative care, but only 2 of them provided clear evidence of cost-effectiveness. Finally, various models showed potential benefits for patients and caregivers and reduction in total healthcare costs; most such evidence pertains to home-based interventions [25].

### Other settings

The remaining evidence relates to the cost-effectiveness of palliative care across multiple settings [20, 21, 25, 37, 44, 54, 61]. A review on [61] palliative care interventions outside the home, identified two studies that included an economic evaluation [67, 68], but only one [68] performed a full cost-effectiveness analysis of the effect of psychosocial support on survival in women with breast cancer. No significant difference in healthcare resource use between the intervention and control groups was found. Smith et al. (2014) [20] identified one cost-effectiveness study on short-term palliative care in multiple sclerosis patients; it suggested potential cost-effectiveness when caregiver burden was used as the main outcome measure [69]. Salamanca et al. (2018) [37] provided inconclusive evidence on the cost-effectiveness of clinical nurse specialists (CNSs): of 13 economic analyses, 7 reported improvement in health economic outcomes, mainly in cardiac patients. CNSs were cost-effective in several countries. In Austria home-based CNSs were both cost-effective and less expensive than standard care for HF patients [70]. In the United Kingdom, CNSs for heart disease patients generated an incremental cost of £13,158 per quality-adjusted life year (QALY) gained compared to the control group [71]. In England CNS intervention improved QoL and reduced readmissions and costs in HF patients [72]. Similarly, a study in the Netherlands CNSs improved health outcomes of patients with severe HF, with a slight increase in costs [73]. Douglas et al. [44] found that CNSs had lower costs and greater benefits than comparator. A review [74] on cost-effectiveness of pain management strategies in advanced cancer found that PainCheck and Tackling Cancer Pain Toolkit [TCPT] were cheaper (respective incremental costs -GBP148 [−EUR168.53] and -GBP474 [−EUR539.74]) and more effective (respective incremental QALYs of 0.010 and 0.013) than usual care.

Two other reviews reported some evidence on the benefits of hospital-based palliative care consultations on patient outcomes, healthcare utilization and cost [54, 75]. Finally, a review by Dixon et al. (2015 [21] found no evidence on cost-effectiveness of ACP.

#### Cost analyses

The following studies reported on the cost and resource use implications of palliative care with various degrees of comprehensiveness. Some were limited to intervention cost while others compared total healthcare costs between an intervention and a control group. Five reviews reported the cost of home-based palliative interventions [31, 76] [24, 59, 60]. Among those, 1 [76] found significant improvement in healthcare use or costs in 16 of 21 (76%) studies that reported those outcomes; the majority of evidence was obtained from quasi-experimental designs. Similarly, Gomes et al. (2013) [59] showed lower costs for home-based group compared with usual care (18 to 35%). Institutional and non-institutional costs, medications costs and in 1 study, informal care costs, were reported. Other reviews found relatively low-quality and inconsistent evidence that home-based interventions might reduce healthcare costs [24, 60, 77], and the costs included varied across studies.

Seven reviews reported the costs of hospital-based interventions [43, 51–57]. Of these, 3 focused on the ICU [51–53], and 1 on the surgical setting [57]. Four reviews [51, 54–56] examined the cost of inpatient palliative care consultations. Specifically, Kyeramateng et al. (2018) [51] showed that palliative care consultations in the ICU reduced LOS and costs, with 5 studies reporting a decrease in ICU cost and 5 others reporting a decrease in total hospital costs. Another study [55] showed that consultation delivered within 3 days of hospitalization may reduce the costs of care, mainly in cancer and multimorbid patients. Similarly, in May et al. (2014) [56], consultation teams were consistently found to be less costly (by 9–25% for hospital costs) compared to usual care, while 1 study estimated a 32% reduction in all healthcare costs over 6 months post discharge. Another study [54] identified some evidence of lower costs in patients receiving consultations. Lilley et al. (2016) [57] found limited evidence regarding interventions to improve access to palliative care (i.e., pain and non-pain symptoms, ACP) in surgical patients. The evidence regarding cost implications of communication interventions was inconclusive [52, 53].

Many studies (*n* = 15) reported mixed findings on the costs of interventions in multiple settings [20, 21, 23, 25, 30–42]. Smith et al. (2014) [20] found consistent evidence that palliative care is less costly relative to comparators, with statistically significant differences in most cases. Four RCTs found lower costs for palliative care. Most studies overlooked out-of-pocket and informal care costs. Salamanca-Balen et al. 2018 [37] found mixed evidence regarding the impact of CNS-led interventions on costs, with 13/46 (28%) studies showing statistically significant cost reductions and 6/46 (13%) studies showing significant cost increases. Bibas et al. 2019 [78] found one CSN-led intervention that observed a significant reduction in costs ($75,850 control vs $51060).

Similarly, Harris et al. (2013) [34] found statistically significant evidence suggesting that palliative care interventions may impact healthcare costs (ranging from a 77% reduction to a 9% cost increase). Seven reviews provided inconclusive evidence regarding cost reduction for specific interventions, including social support [61], CNSs [44], case management [39], communication [41], specialized palliative care [42], palliative and hospice care teams [47], multidisciplinary palliative care interventions [79] and patient and/or caregiver QoL [38].

The evidence regarding the impact of ACP on costs is limited. One review [21] showed savings ranging from USD 64,827 to USD 56,700 in total healthcare costs over 6 months. Klinger et al. (2016) [23] reported net cost savings ranging from USD 1041 to USD 64,830 per patient (relative cost reduction 68 to 5%), with the greatest reductions found for sicker patients. Martin et al. (2016) [62] found limited evidence of impact on costs in 1 study [80] suggesting a significant decrease in both hospital costs and total healthcare costs [81]. Another study [63] identified cost savings for patients with severe dementia who received ACP in the community [82] and for care home residents [80]. Another study [83] on ACP for home health staff reported showed significant reductions in hospitalisation rate, days and healthcare cost.

#### Impact on resource use

Five studies [24, 30–32, 36, 38, 60, 76] examined the impact of home-based interventions on resource use. Bainbridge et al. (2016) [76] showed a significant reduction in healthcare use in the home care group. Other reviews found inconclusive evidence regarding the impact of home-based interventions on resource use, including general healthcare utilization [32], LOS and hospitalizations [24, 31, 38, 60].

Candy et al. (2011) [32] showed that patients receiving hospice care at home had lower healthcare utilization. The strength of this evidence, however, was limited because few of the findings originated from RCTs. Davis et al. (2015) [31] showed that early integration of outpatient and home palliative care may reduce LOS and hospitalizations. In another study [60], it was unclear whether home-based care impacts hospital admissions. Admission to hospital while receiving home-based care varied among trials. One study [38] found that palliative interventions (65%, 11 of 17) reduced hospital use and that home interventions were more effective than other interventions in cancer, COPD and dementia patients.

Evidence regarding the impact of hospital-based palliative care interventions was scarce. One review [58] suggested that hospital-based palliative care may have a positive impact on resource use (i.e., reduced hospitalizations). Two reviews showed a reduction in ICU LOS for intervention patients receiving inpatient palliative care consultations compared with control patients [50, 51]. Two other reviews found that communication interventions may decrease healthcare resource use in multiple dimensions, including duration of mechanical ventilation [53] and hospital and ICU LOS [41, 52, 53]. Waller et al. (2017) [54] found limited evidence that hospital-based interventions improved processes and outcomes, with only 1 study reporting longer hospice stays.

Nine reviews reported inconsistent findings on resource use across multiple settings. Three reviews found limited evidence that palliative care interventions, including case management [20], decreased resource use, [20, 43, 45] and improved hospice use. The evidence on effects of specialized palliative care is sparse, with 3 reviews reporting fewer hospitalizations [22, 33] and higher hospice use [42] in the intervention group. Some evidence showed that CNS might reduce hospitalizations, rehospitalizations/admissions, and LOS [37, 44, 78]. Outpatient palliative care decreased healthcare utilization, including ambulatory care, physician visits, hospitalizations, LOS and readmissions [49]. Datla et al. (2019) [79] reported reduction in health care resource use as a result of multi-disciplinary palliative interventions in patients with symptomatic heart failure.

ACP and palliative care interventions consistently demonstrated a trend toward decreased ICU admissions and reduced ICU and hospital LOS [35, 83]. The mean relative risk reduction in the percentage of admissions with ACP and palliative care consultations was 37%. These findings were supported by two other reviews showing decreased hospitalization rates [62] among patients receiving ACP, including dementia patients [63].

## Discussion

The strongest evidence of cost-effectiveness relates to home-based interventions, suggesting high potential efficiency gains for the health system through a decrease in total healthcare costs and resource use and improvements in patient and caregiver outcomes. The evidence of interventions in other settings was inconsistent. There was some evidence suggesting potential benefits of CNS interventions for patients with heart disease. Overall, the majority of studies focused on cancer patients. One explanation is that disease trajectories for cancer are less heterogeneous than for other diseases [80, 81] and that the end-of-life phase is easier to predict in this patient group [84].

Our findings reveal a potential for reduction in direct healthcare costs and resource use mainly in home-based programs. Inpatient palliative care consultations were consistently associated with fewer hospitalizations, readmissions and reduced costs. We found some evidence of the effectiveness of ACP for reducing resource use. Such interventions have been considered important, especially for dementia patients [85]; however, further research is needed regarding the effectiveness and cost-effectiveness of ACP interventions.

Overall, most studies overlooked important costs, including out-of-pocket, hospice and informal care costs. It is important that future studies systematically report these to get a comprehensive understanding of the health economic implications of palliative and end-of-life care, despite the complexity of their measurement and valuation [86]. Another issue is the lack of consistency in outcomes measurement that hinders comparability across studies. While quality-adjusted life years (QALYs) are widely used in cost-effectiveness, they are limited in their ability to capture the benefits of end-of-life and palliative care. Tools that have been developed to measure wellbeing in the broader sense should be more widely used [87, 88]. More generally, future economic studies in the area should more systematically follow existing economic evaluation guidelines and adopt a societal perspective.

### Strengths and limitations

An important strength of this study is the focus on the economic value of a wide range of interventions. The effectiveness of palliative care interventions has been studied previously, but the focus on cost-effectiveness, resource use and costs has been scarce [25, 89]. By conducting this review of reviews we believe that we have identified the main sources of (high-quality) evidence. This work has several limitations. One key challenge was related to the wide range of settings and intervention types included as well as the diversity of reporting of findings across reviews (e.g., outcome measurement, costing approaches, etc.). While a broad inclusion was a deliberate feature of our study, it made the synthesis of evidence difficult. Most approaches focused on the hospital setting. Hospital costs are known to escalate in the last phase of life, with questionable benefit to patients [90, 91]. Furthermore, the evidence is subject to a range of methodological limitations as discussed above. Also, as evidence often comes from retrospective data important information (e.g., patient preferences) were not be available [92].

## Conclusions

Home-based palliative care may contribute to a dual improvement in quality of care by reducing aggressive medicalization end-of-life and concomitantly reducing costs. Hospital-based palliative care interventions may improve patient outcomes, healthcare utilization and costs. Evidence regarding other approaches is less conclusive. This study provides a foundation for discussions between policy makers and clinical services managers regarding resource allocation and the commissioning of palliative care services. There is a need for greater consistency in costs and outcome measures reporting, including breadth of capture (e.g., informal care costs, hospice costs, better-suited measure of patient outcomes, etc.). In addition, as RCTs are rarely feasible in the area, a particular focus on the quality of observational, and quasi-experimental evidence, is warranted. On the clinical side, further examination of who can deliver interventions is important for clinical practice – can generalists (non palliative care specialists) deliver these interventions as well as specialists? What are the enablers and barriers to successful interventions? Also, information on timing in relation to death would be useful to understand more fully the impact and resource implications of palliative care interventions. Research that addresses these practical issues as well as examining effectiveness and cost would help policy makers and clinical teams know where to invest resources. Better patient care can be provided at lower costs for people in the last phase of life with intentional palliative care interventions.

## Data Availability

Data sharing is not applicable to this article as no datasets were generated or analysed during the current study.

## Abbreviations

ACP: Advance care planning
CNS: Clinical nurse specialist interventions
ICU: Intensive care unit
LOS: Length of stay
QOL: Quality of life
